# Tislelizumab-induced distal renal tubular acidosis presenting with life-threatening hypokalemia: a case report

**DOI:** 10.3389/fimmu.2026.1848498

**Published:** 2026-06-11

**Authors:** Jiaju Xu, Lidong Qin, Ping Wang, Zhen Liu, Lili Yang, Hongwei Zhang, Yanqiu Wang, Jiao Gao, Meishu Zhao, Kaili Zhang

**Affiliations:** Department of Medical Oncology, Tai’an City Central Hospital, Tai’an, Shandong, China

**Keywords:** distal renal tubular acidosis, hypokalemia, immune checkpoint inhibitors, immune-related adverse events, tislelizumab

## Abstract

Immune checkpoint inhibitors (ICIs) can cause immune-related adverse events (irAEs) affecting multiple organs. Distal renal tubular acidosis (dRTA) as a renal irAE is exceedingly rare, often delaying diagnosis due to nonspecific symptoms. A 63-year-old man with stage IIIA lung squamous cell carcinoma developed recurrent nausea and vomiting after three cycles of nab-paclitaxel/carboplatin combined with tislelizumab. Laboratory evaluation revealed profound metabolic acidosis (CO_2_-CP nadir 11.6 mmol/L) and life-threatening hypokalemia (serum potassium nadir 1.1 mmol/L), requiring ICU admission and mechanical ventilation. Despite aggressive intravenous potassium supplementation (up to 20 mEq/h), hypokalemia paradoxically worsened. Urine pH was 7.0 during systemic acidosis, indicating impaired renal acidification. After excluding other causes, a diagnosis of tislelizumab-induced dRTA was established. Tislelizumab was discontinued, and treatment with oral potassium citrate and prednisone (60 mg/day, tapered over 5.5 months) led to complete resolution of metabolic abnormalities. Over one year of follow-up, the patient has maintained normal renal function without recurrence and achieved a major partial response of his lung cancer. This first reported case of tislelizumab-induced dRTA highlights the need for vigilance when encountering unexplained, potassium-refractory electrolyte disturbances during ICI therapy, emphasizing early diagnosis, drug discontinuation, and pathophysiology-guided therapy.

## Introduction

Renal tubular acidosis (RTA) encompasses disorders characterized by impaired renal acid excretion, leading to normal anion gap metabolic acidosis (1). Type 1, or distal renal tubular acidosis (dRTA), results from defective hydrogen ion secretion in the distal tubule, preventing normal urinary acidification (urine pH persistently >5.5 during systemic acidosis) (1). Clinically, dRTA presents with hyperchloremic metabolic acidosis and refractory hypokalemia, which can lead to muscle weakness, cardiac arrhythmias, and respiratory muscle paralysis (2). Secondary dRTA is most commonly associated with autoimmune diseases and certain drugs (2).

Immune checkpoint inhibitors (ICIs) targeting PD-1/PD-L1 have revolutionized cancer treatment but can trigger immune-related adverse events (irAEs) resembling autoimmune disorders (3, 4). Renal irAEs occur in approximately 2-5% of patients, with acute interstitial nephritis (AIN) being the most common manifestation (5). However, selective impairment of tubular function—particularly acidification—leading to dRTA is extremely rare, with only isolated case reports to date (6, 7).

Clinical symptoms such as fatigue and nausea are often misattributed to tumor progression or chemotherapy, delaying diagnosis. In our patient, the serum potassium nadir of 1.1 mmol/L and poor response to conventional potassium supplementation represent an extreme phenotype that provides unique insights into ICI-mediated tubular injury. We present this critically ill case to raise awareness of this rare but life-threatening irAE.

## Case presentation

### Initial presentation and anti-tumor therapy

A 63-year-old man was diagnosed in April 2024 with moderately differentiated lung squamous cell carcinoma of the right upper lobe (cT2aN2aM0, stage IIIA). He had no significant medical history; baseline laboratory values were normal. He received three cycles of nab-paclitaxel (400 mg)/carboplatin (400 mg) plus tislelizumab (200 mg) (April 13, May 6, and June 11, 2024), achieving partial response.

### Onset and progression of symptoms

Ten days after the third cycle, the patient developed progressive nausea, vomiting, and fatigue. Laboratory tests on June 1, 2024, showed potassium 2.95 mmol/L, CO_2_−CP 19.6 mmol/L, and creatinine 120 μmol/L. Despite intravenous potassium supplementation, symptoms persisted, and by July 13, he presented with severe nausea, vomiting, and weakness. Labs: potassium 1.85 mmol/L, chloride 107.0 mmol/L, CO_2_−CP 11.6 mmol/L, anion gap 18.4, creatinine 147 μmol/L, urine pH 7.0. ECG showed U waves and prolonged QT interval.

Initial resuscitation included intravenous 0.9% normal saline at a rate of 100–150 mL/h, along with potassium chloride (up to 20 mEq/h) and sodium bicarbonate (50 mEq over 2 hours, then 25 mEq/h). Despite aggressive intravenous potassium and sodium bicarbonate, his condition worsened. Nineteen hours later, potassium dropped to 1.1 mmol/L, with CO_2_−CP 17.3 mmol/L and creatinine 173 μmol/L. He developed confusion and type II respiratory failure, requiring intubation and mechanical ventilation. Arterial blood gas confirmed profound metabolic acidosis (pH 6.967) and severe hypokalemia (K^+^ 1.29 mmol/L).

### Diagnostic evaluation

Refractory hypokalemia, normal anion gap metabolic acidosis, and alkaline urine (pH 7.0) during systemic acidosis suggested dRTA. Multidisciplinary team (MDT) excluded gastrointestinal losses and other drugs, including proton pump inhibitors (PPIs), analgesics, antibiotics, and tenofovir. The patient had no PPI exposure during the period of ICI therapy and dRTA onset. (Of note, pantoprazole was briefly used at 40 mg intravenously on the day of each of the subsequent three chemotherapy cycles after dRTA resolution, for prophylaxis of chemotherapy-induced gastrointestinal symptoms; this occurred after the acid-base and electrolyte disturbances had already improved, and does not affect causality.) Renal biopsy was declined due to critical condition and bleeding risk. The Naranjo score for drug causality was 6, indicating a probable relationship (8). Findings met diagnostic criteria for type 1 dRTA (9). MDT consensus: tislelizumab-induced acute tubulointerstitial nephritis with dRTA.

**Figure 1:**
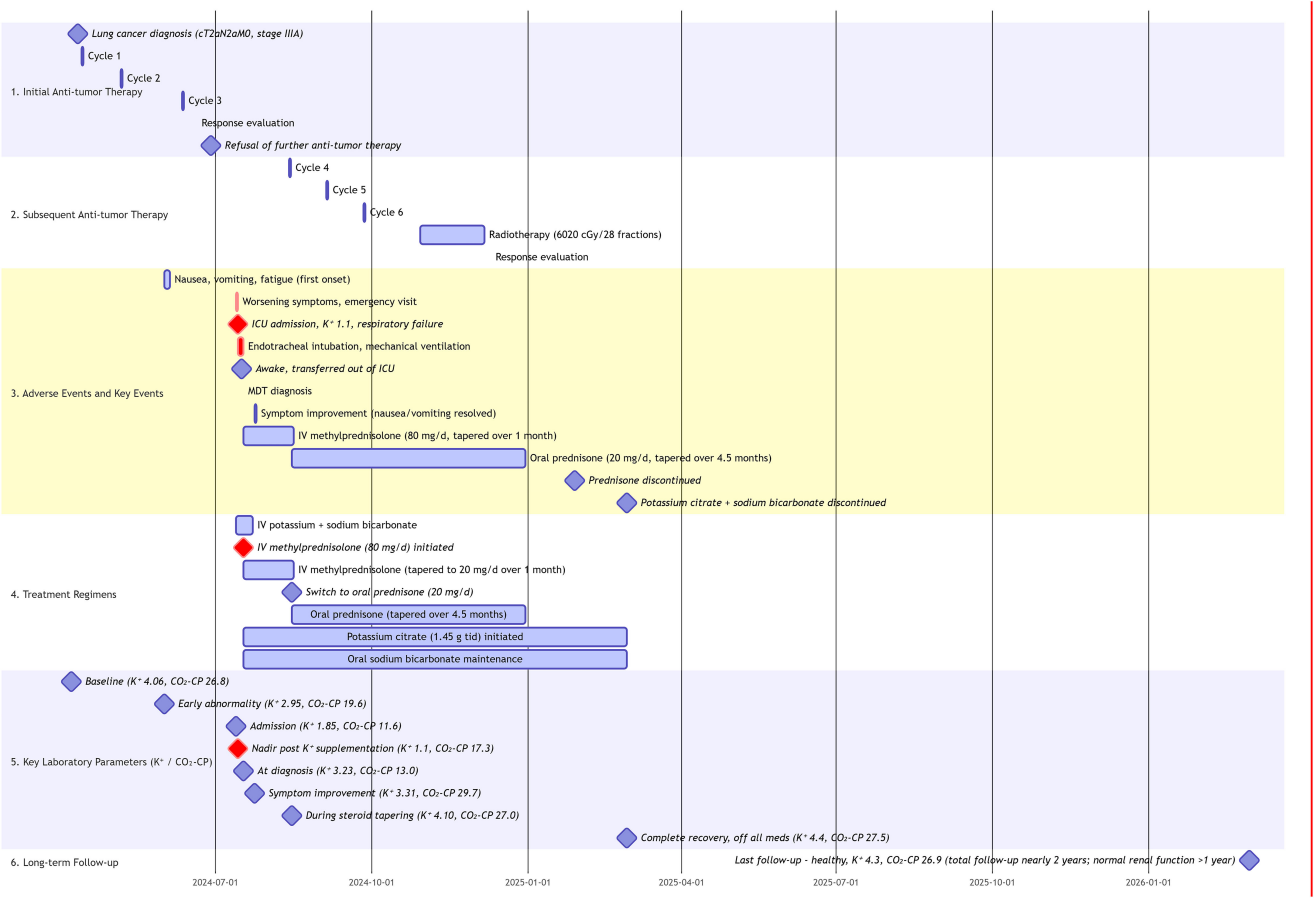
Complete diagnostic and therapeutic timeline. The timeline spans from April 2024 to March 2026 and is organized into six domains: (1) initial anti-tumor therapy (cycles 1–3 with nab-paclitaxel/carboplatin plus tislelizumab); (2) subsequent anti-tumor therapy (cycles 4–6 chemotherapy alone and radiotherapy); (3) adverse events and key clinical events; (4) treatment regimens; (5) key laboratory parameters (serum potassium [K^+^] and carbon dioxide combining power [CO_2_-CP]); and (6) long-term follow-up. Milestones are depicted as diamonds, while continuous interventions are shown as horizontal bars. Key milestones include: • April 11, 2024: Lung cancer diagnosis (cT2aN2aM0, stage IIIA). • April–June 2024: Three cycles of tislelizumab-containing chemotherapy (partial response). • June 1, 2024: Initial symptoms (nausea, fatigue, hypokalemia 2.95 mmol/L). • July 14, 2024: Critical deterioration (K^+^ 1.1 mmol/L), ICU admission, intubation. • July 17, 2024: MDT diagnosis of dRTA; initiation of methylprednisolone, potassium citrate, and sodium bicarbonate. • July 24, 2024: Symptom improvement (K^+^ 3.31 mmol/L, CO_2_-CP 29.7 mmol/L). • August–September 2024: Three cycles of chemotherapy (nab-paclitaxel + carboplatin, without tislelizumab). • October–December 2024: Radiotherapy (6020 cGy/28 fractions). • December 10, 2024: Post-treatment evaluation: major partial response. • January 28, 2025: Prednisone discontinued. • February 28, 2025: Potassium citrate and sodium bicarbonate discontinued; complete normalization of electrolytes (K^+^ 4.4 mmol/L, CO_2_-CP 27.5 mmol/L). • March 2026: Last follow-up (over one year), healthy with normal renal function. Abbreviations: dRTA, distal renal tubular acidosis; ICI, immune checkpoint inhibitor; ICU, intensive care unit; MDT, multidisciplinary team; PR, partial response; CO_2_-CP, carbon dioxide combining power; nab-paclitaxel, albumin-bound paclitaxel.

The timeline is shown in **Figure 1**. Serial laboratory parameters are detailed in **Table 1**; the complete blood gas sequence during ICU stay is provided in **Table 2**.

**Table 1:** Dynamic changes in key laboratory parameters (venous blood and urine pH) throughout the treatment course.

Treatment stage	K^+^ (3.5-5.0 mmol/L)	Cl^-^ (96–106 mmol/L)	CO_2_-CP (22–29 mmol/L)	AG (8–16 mmol/L)	Cr (59-104 μmol/L)	Urine pH (4.50-8.00)	Main clinical manifestations and pathophysiological changes
Baseline (2024-04-07)	4.06	106.6	26.8	8.1	75.0	6.0	Normal
Post 3 Cycles Chemoimmunotherapy (2024-06-14)	2.95	105.1	19.6	9.1	157	5.4	Recurrent nausea and vomiting; mild hypokalemia with normal anion gap metabolic acidosis.
Admission (2024-07-14 00:16)	1.85	107.0	11.6	18.4	147	7	Worsening nausea, vomiting, fatigue, and poor appetite. Severe hypokalemia with metabolic acidosis. Inappropriately alkaline urine (pH 7.0) despite systemic acidemia–key diagnostic clue for dRTA.
Post K^+^ Supplementation (19h later) (2024-07-14 20:23)	1.10	108.0	17.3	19.7	173	7.2	Paradoxical decrease in serum potassium despite aggressive IV K^+^ supplementation (up to 20 mEq/h), indicating ongoing renal potassium wasting and severe metabolic acidosis.
At dRTA Diagnosis (2024-07-17)	3.23	113	13.0	13.0	93	7.4	Persistent fatigue, nausea, and vomiting; mild hypokalemia with refractory metabolic acidosis. MDT confirmed dRTA diagnosis.
Post Potassium Citrate and Corticosteroid Therapy (2024-08-12)	3.31	100.9	29.7	12.2	75	7.0	Systemic acidosis has been corrected, but renal tubular acidification function has not yet recovered — Potassium citrate corrects systemic acidosis by replenishing alkali reserves. However, despite ongoing corticosteroid therapy, the distal tubular hydrogen ion secretion defect persists at this time point, indicating that functional recovery of the tubules lags behind metabolic correction.
Post Potassium Citrate and Corticosteroid Therapy (2024-09-03)	4.10	100.6	27.0	14.2	84	6.5	Renal tubular function begins to recover after sustained corticosteroid therapy — Immune-mediated inflammation is suppressed, and the distal tubules gradually restore hydrogen ion secretion capacity, leading to a decrease in urine pH.
Off All Medications (Clinical Cure) (2025-02-28)	4.4	100.4	27.5	14.2	74	6.0	Complete recovery; all medications discontinued.
Last Follow-up (2026-03-01)	4.3	105.4	26.9	10.9	88	6.0	Last follow-up: patient healthy, asymptomatic, no electrolyte abnormalities (follow-up nearly 2 years)

Reference ranges are shown in parentheses. The serum potassium decreased from 1.85 mmol/L on admission to 1.1 mmol/L after intravenous potassium supplementation, a paradoxical phenomenon characteristic of dRTA. This occurs because severe metabolic acidosis inhibits intracellular potassium shift, and ongoing renal potassium wasting due to distal tubular dysfunction overwhelms the supplementation.

CO_2_-CP, carbon dioxide combining power; AG, anion gap; Cr, creatinine; MDT, multidisciplinary team; dRTA, distal renal tubular acidosis.

**Table 2:** Serial arterial blood gas analysis during ICU stay.

Time point	pH (7.350-7.450)	PCO_2_ (32.0-48.0 mmHg)	HCO_3_^-^ (mmol/L)	BE (-3.0~+3.0 mmol/L)	K^+^ (3.50-5.10 mmol/L)	Cl^-^ (98.0-107.0 mmol/L)	AG (8.0-16.0 mmol/L)
2024-07-14 22:23	6.967	91.6	20.4	-12.35	1.29	113.6	12.9
2024-07-14 23:56	7.141	51.9	17.3	-11.49	1.33	118.2	13.0
2024-07-15 01:51	7.171	46.1	16.4	-11.63	1.59	121.5	12.6
2024-07-15 04:12	7.234	40.7	16.8	-10.05	1.70	123.3	11.3
2024-07-15 07:04	7.269	31.9	14.3	-11.55	2.09	126.5	13.3
2024-07-15 08:41	7.250	33.5	14.1	-12.19	2.09	126.5	13.0
2024-07-15 10:31	7.264	33.4	13.8	-12.07	2.03	129.6	12.8
2024-07-15 22:22	7.263	27.6	12.2	-13.49	2.58	132.6	10.6
2024-07-16 02:23	7.276	26.1	11.9	-13.5	3.61	133.2	10.6
2024-07-16 04:31	7.277	26.4	12.0	-13.38	3.71	133.8	10.2
2024-07-16 06:16	7.282	26.5	12.2	-13.08	4.39	134.4	10.8
2024-07-16 09:06	7.316	24.8	12.4	-12.36	3.16	131.8	11.0

Reference ranges are shown in parentheses. Values outside the normal range are highlighted in bold. BE, base excess; AG, anion gap.

Key Dynamic Changes (as of 09:06 on July 16):

• pH gradually improved from a nadir of 6.967 to 7.316, indicating active correction of acidosis, though still below the normal range (>7.35).

• Serum potassium (K^+^) recovered to the normal range (4.39 mmol/L at 06:16) but showed a slight decrease to 3.16 mmol/L at 09:06, highlighting the need for continuous adjustment of potassium supplementation.

• Bicarbonate (HCO_3_^-^) remained significantly low (12–12.4 mmol/L), far below the normal level (>22 mmol/L), suggesting insufficient alkali supplementation for dRTA or unrecovered renal acidification function.

• Hyperchloremia persisted, with Cl^-^ consistently above 130 mmol/L, a typical feature of normal anion gap hyperchloremic metabolic acidosis in dRTA.

• Anion gap (AG) remained within the normal range (10–13 mmol/L), ruling out superimposed high anion gap metabolic acidosis.

### Treatment and outcome

Upon diagnosis, we implemented the following measures: 1) discontinuation of tislelizumab; 2) withdrawal of all potentially nephrotoxic drugs; 3) high-dose methylprednisolone (initiated on July 17, 2024) to suppress immune-mediated inflammation; 4) oral potassium citrate (1.45 g three times daily) to correct acidosis and hypokalemia; and 5) oral sodium bicarbonate for sustained alkalinization. Symptoms markedly improved within one week (July 24, 2024): serum potassium rose to 3.31 mmol/L, CO_2_−CP 29.7 mmol/L. Methylprednisolone was transitioned to oral prednisone on August 15 and tapered over 5.5 months until discontinuation on January 28, 2025, while potassium citrate and sodium bicarbonate were continued.

The patient subsequently completed three chemotherapy cycles (nab-paclitaxel + carboplatin on August 13, September 4, September 26, 2024) and radiotherapy (6020 cGy/28 fractions, October 29–December 5, 2024), achieving major partial response.

On February 28, 2025, all electrolytes normalized (K^+^ 4.4 mmol/L, CO_2_−CP 27.5 mmol/L); potassium citrate and sodium bicarbonate were discontinued. As of March 2026, the patient remains healthy with normal renal function for over one year.

## Discussion

### Literature review and positioning

ICI-related dRTA is an extremely rare irAE. To our knowledge, only a handful of cases have been reported, including three by Herrmann et al. (6) using PD-1 inhibitors (nivolumab/pembrolizumab), one by Qiu et al. (7) using sintilimab (another PD-1 inhibitor), and a few involving CTLA-4 inhibitors or combination therapy (10). Beyond initial case reports, the question of ICI rechallenge has been addressed in a recent report (11). A summary of previously reported cases is provided in **Supplementary Table 1**.

We acknowledge the absence of histopathological confirmation as a limitation. However, Herrmann et al. demonstrated loss of key acid-base transporters (H^+^-ATPase and AE1) in renal biopsies from ICI-related dRTA patients, providing a pathological correlate (6). Our patient’s rapid response to corticosteroids supports a similar immune-mediated mechanism, and the extreme phenotype (K^+^ 1.1 mmol/L) uniquely complements prior histopathological observations.

Several key observations emerge from the literature: (1) PD-1/PD-L1 inhibitors are the predominant causative agents; (2) clinical severity varies widely; and (3) most patients respond favorably to corticosteroids. Notably, our case represents the lowest reported serum potassium in ICI-associated dRTA and required a protracted corticosteroid taper (5.5 months) for sustained remission. Thus, this case expands the literature and provides insights into the extreme phenotype and management.

### Mechanistic discussion

The pathogenesis of ICI-related dRTA is likely immune-mediated. ICIs disrupt peripheral immune tolerance, which can inadvertently lead to cross-recognition of self-antigens expressed on normal tissues (12, 13).

Distal tubular α-intercalated cells express unique functional proteins, including vacuolar H^+^-ATPase for proton secretion and anion exchanger 1 (AE1) for bicarbonate reclamation (14, 15). In autoimmune diseases such as Sjögren’s syndrome, T-cell attacks against these proteins directly cause dRTA (16). ICIs may trigger a similar autoimmune response against these same targets (13, 17). Herrmann et al. provided direct histological evidence by demonstrating markedly reduced expression of these acidification proteins in renal biopsies from ICI-treated patients (6).

**Figure 2:**
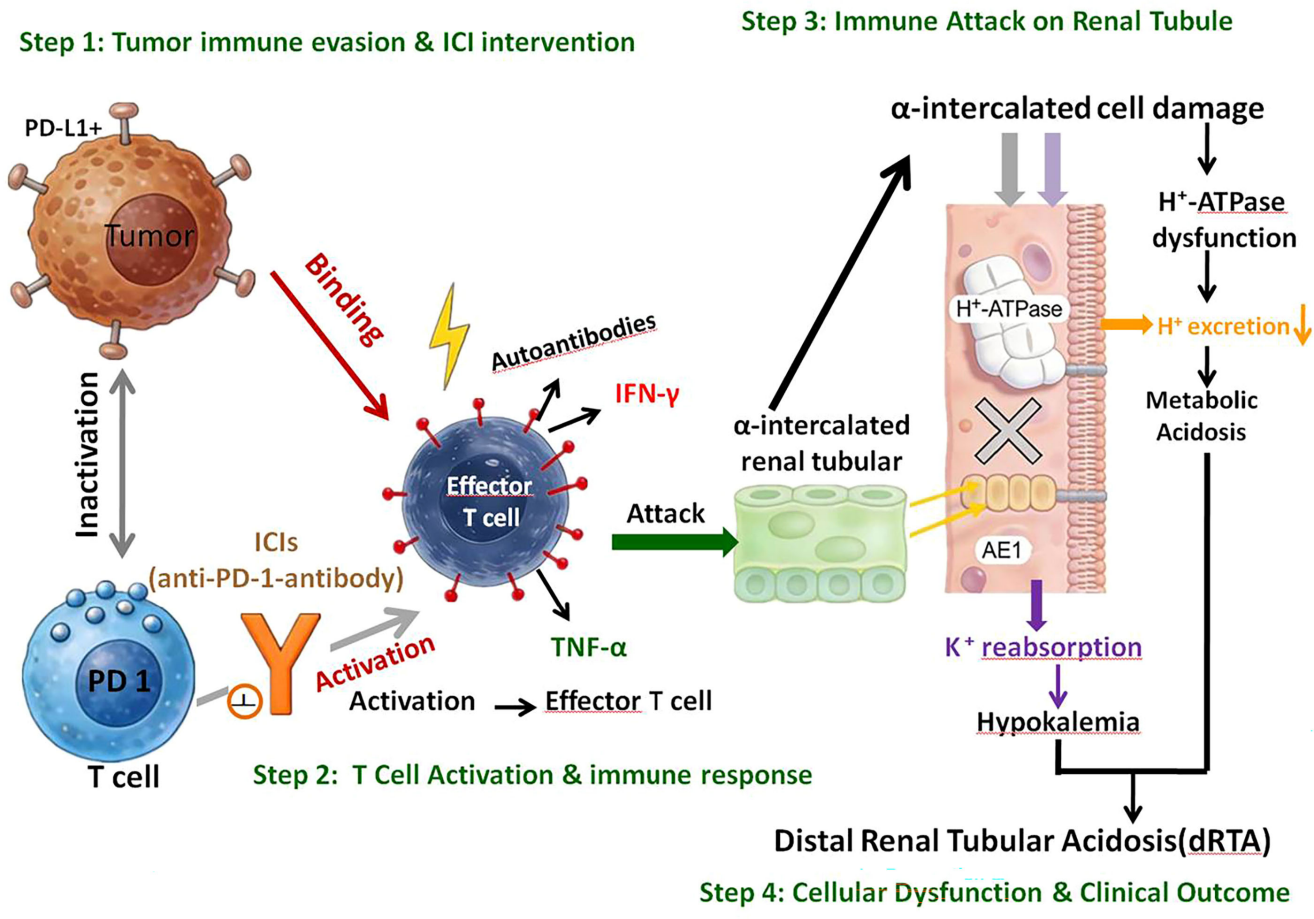
Schematic diagram illustrating the potential mechanism of immune checkpoint inhibitor-induced distal renal tubular acidosis (dRTA) complicated with hypokalemia. (Step1) Tumor immune evasion and ICI intervention. Tumor cells expressing PD−L1 bind to PD−1 on T cells, inducing T cell inactivation and immune evasion. Immune checkpoint inhibitors (ICIs, e.g., anti−PD−1 antibodies) block this interaction, leading to T cell activation and differentiation into effector T cells. (Step2) T cell activation and immune response. Activated effector T cells secrete pro−inflammatory cytokines (e.g., IFN−γ, TNF−α) and may cooperate with autoantibodies, contributing to systemic immune activation. (Step3) Immune−mediated attack on renal tubules. Activated T cells and cytokines target distal tubular α−intercalated cells, which express unique functional proteins including vacuolar H^+^−ATPase (responsible for proton secretion) and anion exchanger 1 (AE1, responsible for bicarbonate reclamation). Immune−mediated injury leads to dysfunction or loss of these transporters. (Step4) Cellular dysfunction and clinical outcome. Impaired H^+^−ATPase function reduces proton excretion, causing metabolic acidosis. Concurrent AE1 dysfunction disrupts bicarbonate reabsorption and alters potassium handling, leading to renal potassium wasting and hypokalemia. These combined defects result in the characteristic features of dRTA: hyperchloremic metabolic acidosis, inappropriately alkaline urine, and hypokalemia. Abbreviations: ICI, immune checkpoint inhibitor; PD−1, programmed death−1; PD−L1, programmed death ligand−1; IFN−γ, interferon−gamma; TNF−α, tumor necrosis factor−alpha; AE1, anion exchanger 1; dRTA, distal renal tubular acidosis.

Additionally, adenosine via A1 receptors modulates renal tubular ion transport, including H^+^-ATPase activity (18). PD-1/PD-L1 signaling might indirectly influence acidification by interfering with paracrine pathways such as adenosine, a mechanism warranting further investigation. Finally, concurrent carboplatin, with its nephrotoxic potential, may amplify the ICI-induced autoimmune reaction and contribute to the exceptional severity in our patient. Importantly, although PPIs have been reported to potentiate H^+^-ATPase dysfunction in some settings, our patient was not exposed to PPIs during the relevant period, excluding this confounding factor. (As noted in the Case Report, pantoprazole was used briefly after dRTA resolution for chemotherapy support, which does not affect causality.) A schematic representation of this proposed mechanism is provided in **Figure 2**.

### Clinical implications

#### Diagnostic clues

A key observation was the paradoxical decline in serum potassium despite aggressive supplementation (from 1.85 to 1.1 mmol/L over 19 hours). This phenomenon has a clear pathophysiological basis: severe metabolic acidosis promotes intracellular shift of hydrogen ions in exchange for potassium, while impaired distal acidification causes ongoing urinary potassium wasting. When acidosis is partially corrected, hydrogen ions move out and potassium re-enters cells, but renal potassium loss continues unabated. Although dilution from intravenous fluids might have contributed marginally, the dominant mechanisms are the intracellular shift of potassium upon acidosis correction and ongoing renal potassium wasting. Clinicians encountering unexplained hypokalemia in ICI-treated patients should be alerted by this paradoxical response and promptly investigate with urine pH.

A simple heuristic: ICI therapy + unexplained refractory hypokalemia + normal AG metabolic acidosis = ICI-related dRTA until proven otherwise.

#### Therapeutic strategy

Resistance to potassium chloride stems from extracellular acidosis inhibiting intracellular potassium shift. Thus, the key is not merely potassium replacement but correction of acidosis. Potassium citrate, an alkalinizing agent, is metabolized to bicarbonate, simultaneously replenishing potassium and correcting acidosis while alkalinizing urine to prevent calcium precipitation.

For suspected immune-mediated injury, early corticosteroid administration is essential to halt disease progression. The prolonged taper in our case, although not standardized, successfully prevented relapse and provides a reference for managing severe dRTA.

#### Multidisciplinary approach

This case underscores the value of a multidisciplinary team (MDT), as every step benefited from collective expertise across specialties.

#### Rechallenge considerations

Shah and Weiner reported successful rechallenge with a different PD-1 inhibitor without recurrence of dRTA (11), suggesting that ICI-associated dRTA may be drug-specific rather than a class effect. As noted in ICI-associated acute kidney injury (19), rechallenge decisions must balance antitumor efficacy against toxicity risk. In our patient, given the life-threatening severity, we elected to permanently discontinue all ICIs at present. However, for patients with milder presentations or limited therapeutic options, as well as for this patients if disease progression occurs in the future, switching to a different ICI may be considered after thorough risk-benefit discussion.

### Strengths and limitations

Strengths include comprehensive documentation of the clinical course with detailed ICU data and over one year of follow-up. Limitations include the absence of histopathological confirmation (biopsy declined) and lack of serial urine pH measurements during follow-up, limiting understanding of tubular recovery dynamics. Furthermore, urine electrolytes for calculation of the urine anion gap were not obtained in this case. Although our institution can perform such measurements on 24-hour urine specimens, we did not collect the appropriate sample, partly due to the urgency of the critical illness (K^+^ 1.1 mmol/L, ICU admission) and partly due to a lack of awareness at the time. This represents an operational and educational limitation. However, the diagnosis of dRTA was securely established by the combination of hyperchloremic metabolic acidosis, inappropriately alkaline urine (pH 7.0), and exclusion of gastrointestinal losses. This case underscores the importance of remembering to obtain urine electrolytes (ideally from a 24-hour collection or at least a spot urine) when dRTA is suspected, as the urine anion gap provides supportive evidence.

## Conclusion

We report the first case of tislelizumab-induced distal renal tubular acidosis presenting with extreme hypokalemia (1.1 mmol/L) and respiratory failure. With over one year of sustained follow-up, the patient has maintained normal renal function and electrolyte balance without recurrence, confirming the durability of remission. This case highlights the need for heightened vigilance when encountering unexplained, potassium-refractory electrolyte disturbances during ICI therapy. Early recognition, permanent discontinuation of the offending agent, and pathophysiology-guided therapy with potassium citrate and corticosteroids are key to improving outcomes.

## Data Availability

The original contributions presented in the study are included in the article/**Supplementary Material**. Further inquiries can be directed to the corresponding author.
