# Supplementary material for: Tislelizumab-induced distal renal tubular acidosis presenting with life-threatening hypokalemia: a case report
Source: Front Immunol. 2026 Jun 11;17:1848498. doi: 10.3389/fimmu.2026.1848498 (PMC13294038; doi:10.3389/fimmu.2026.1848498)
Supplement: Supplementary file 1 [file Table1.docx]

**Supplementary Table S1.** Literature Review: Summary of Reported Cases of ICI-Related dRTA

| First Author/Year | Tumor Type / ICI Agent | Concurrent Medication / Time to Onset | Nadir Electrolytes (K⁺/HCO₃⁻, mmol/L) | Treatment and Outcome |
| --- | --- | --- | --- | --- |
| El Bitar S, 2018^[^[^1^](#_ENREF_1)^]^ | Non-small cell lung cancer / Nivolumab | None, approximately 8 weeks | 2.4/11 | Dexamethasone + IV sodium bicarbonate, followed by oral bicarbonate and gradual prednisone tapering → Improved |
| Charmetant X, 2019^[^[^2^](#_ENREF_2)^]^ | Lung adenocarcinoma / Nivolumab | Cisplatin + pemetrexed, after 6 cycles | 3.3/11 | Alkali supplementation ineffective; corticosteroids 1 mg/kg/day → Improved |
| Herrmann SM,  2020^[^[^3^](#_ENREF_3)^]^ | Case 1: Lung adenocarcinoma / Pembrolizumab Case 2: Malignant melanoma / Nivolumab Case 3: Renal cell carcinoma / Nivolumab | Case 1: Carboplatin, 18 weeks  Case 2: None, 16 months  Case 3: None, 2 years | Case 1: 2.8/17.0  Case 2: 4.2/15.0  Case 3: 3.9/11.0 | Case 1: Oral prednisone + sodium bicarbonate + K⁺ supplementation→ Improved  Case 2: Potassium citrate + prednisone → Improved  Case 3: Prednisone → Improved |
| Atiq SO, 2021^[^[^4^](#_ENREF_4)^]^ | Cutaneous squamous cell carcinoma / Pembrolizumab | None, approximately 10 weeks | 2.6/15 | Oral prednisone 1 mg/kg/day (slow tapering over ~6 weeks) + aggressive K⁺/alkali supplementation → Improved |
| Doodnauth AV, 2021^[^[^5^](#_ENREF_5)^]^ | Malignant melanoma / Nivolumab + Ipilimumab | None, approximately 12 weeks (after cycle 4) | 2.4/9.0 | Prednisone 1 mg/kg/day (gradual tapering) + K⁺/alkali supplementation → Improved |
| Qiu X, 2023^[^[^6^](#_ENREF_6)^]^ | Metastatic cardiac carcinoma / Sintilimab | Oxaliplatin + nab-paclitaxel, approximately 3 months (after 4th cycle) | 2.8/9.4 | Sintilimab and chemotherapy discontinued; sodium bicarbonate + potassium citrate → Improved (no corticosteroids used) |
| Shah CV, 2023^[^[^7^](#_ENREF_7)^]^ | Tonsillar squamous cell carcinoma / Pembrolizumab | Carboplatin + paclitaxel, approximately 3 months | 3.2/15.0 | Oral potassium citrate alone, no corticosteroids → Improved |
| Fujioka H, 2024^[^[^8^](#_ENREF_8)^]^ | Mandibular gingival cancer / Pembrolizumab | Cisplatin + fluorouracil, approximately 4 weeks | 2.8/14.7 | Methylprednisolone pulse (500 mg/day × 3 days) → oral prednisone (starting 50 mg/day, tapered) → Improved |

Note: All patients demonstrated clinical and biochemical improvement following treatment. The patient reported by Shah et al. ^[^[^7^](#_ENREF_7)^]^ (tonsillar squamous cell carcinoma / pembrolizumab) was later rechallenged with nivolumab after disease progression and did not develop recurrent dRTA during 8 months of follow-up ^[^[^9^](#_ENREF_9)^]^.

ICI, immune checkpoint inhibitor; dRTA, distal renal tubular acidosis.

**References**

J

1 El Bitar S, Weerasinghe C, El-Charabaty E, Odaimi M. Renal Tubular Acidosis an Adverse Effect of PD-1 Inhibitor Immunotherapy. *Case reports in oncological medicine* 2018; **2018**: 8408015 [PMID: 29666732 PMCID: 5831873 DOI: 10.1155/2018/8408015]

2 Charmetant X, Teuma C, Lake J, Dijoud F, Frochot V, Deeb A. A New Expression of Immune Checkpoint Inhibitors’ Renal Toxicity: when Distal Tubular Acidosis Precedes Creatinine Elevation. *Clinical kidney journal* 2019; **13**(1): 42-45 [DOI: 10.1093/ckj/sfz051]

3 Herrmann SM, Alexander MP, Romero MF, Zand L. Renal Tubular Acidosis and Immune Checkpoint Inhibitor Therapy: An Immune-Related Adverse Event of PD-1 Inhibitor-A Report of 3 Cases. *Kidney medicine* 2020; **2**(5): 657-662 [PMID: 33089143 PMCID: 7568062 DOI: 10.1016/j.xkme.2020.05.015]

4 Atiq SO, Gokhale T, Atiq Z, Holmes R, Sparks M. A CASE OF PEMBROLIZUMAB INDUCED DISTAL RENAL TUBULAR ACIDOSIS. *Journal of general internal medicine* 2021; **36**(SUPPL 1) [DOI: 10.1177/2399369321992776]

5 Doodnauth AV, Klar MM, Malik ZR, Patel KH, McFarlane SI. A Rare Presentation of Checkpoint Inhibitor Induced Distal RTA. *Case reports in oncological medicine* 2021; **2021**: 7406911 [PMID: 34327031 PMCID: 8310435 DOI: 10.1155/2021/7406911]

6 !!! INVALID CITATION !!!

7 Shah CV, Lee HW, Clapp WL, Weiner ID. A Novel Form of Renal Tubular Acidosis Associated With Immune Checkpoint Inhibitors. *Kidney international reports* 2023; **8**(1): 197-201 [PMID: 36644354 PMCID: 9831939 DOI: 10.1016/j.ekir.2022.10.019]

8 Fujioka H, Kakeshita K, Imamura T, Arisawa Y, Yokoyama S, Yamazaki H, Koike T, Minamisaka T, Hirabayashi K, Kinugawa K. Pembrolizumab-induced Acute Tubulointerstitial Nephritis Accompanying Fanconi Syndrome and Type 1 Renal Tubular Acidosis. *Internal medicine* 2024; **63**(4): 533-539 [PMID: 37380456 PMCID: 10937132 DOI: 10.2169/internalmedicine.1918-23]

9 Shah CV, Weiner ID. Rechallenge in Immune Checkpoint Inhibitors Associated Renal Tubular Acidosis. *Kidney international reports* 2023; **8**(7): 1476-1477 [PMID: 37441465 PMCID: 10334382 DOI: 10.1016/j.ekir.2023.05.013]
